# The pain, agitation, and delirium practice guidelines for adult critically ill patients: a post-publication perspective

**DOI:** 10.1186/2110-5820-3-9

**Published:** 2013-04-02

**Authors:** Yoanna Skrobik, Gerald Chanques

**Affiliations:** 1 Soins Intensifs, Hôpital Maisonneuve Rosemont, Montréal, QC H1T 2M4, Canada; 2 Intensive Care and Anaesthesiology Department (DAR), Saint Eloi Hospital, Montpellier University Hospital, 80, Avenue Augustin Fliche, Montpellier cedex 5, 34295, France

## Abstract

The recently published Clinical Practice Guidelines for the Management of Pain, Agitation, and Delirium in Adult Patients in the Intensive Care Unit differ from earlier guidelines in the following ways: literature searches were performed in eight databases by a professional librarian; psychometric validation of assessment scales was considered in their recommendation; discrepancies in recommendation votes by guideline panel members are available in online supplements; and all recommendations were made exclusively on the basis of evidence available until December of 2010. Pain recognition and management remains challenging in the critically ill. Patient outcomes improve with routine pain assessment, use of co-analgesics and administration as well as dose adjustment of opiates to patient needs. Thoracic epidurals help ease patients undergoing abdominal aortic surgery. Little data exists to guide clinicians as to the type or dose of co-analgesics; no opiate choice is associated with better patient outcomes. Lighter or no sedation is beneficial, and interruption is desirable in patients who require deep sedation for specific pathologic states. Delirium screening is probably useful; no treatment modality can be unequivocally recommended, and the benefit of prophylaxis is established only for early mobilization. The details of these recommendations, as well as more recent publications that complement the guidelines, are provided in this commentary.

## Review

### Introduction

The Clinical Practice Guidelines for the Management of Pain, Agitation, and Delirium in Adult Patients in the Intensive Care Unit was recently published [1]. This commentary summarizes the guidelines process, its efforts to ensure transparency and scientific rigor as well as describing some areas that remain controversial. How these guidelines differ from earlier versions, knowledge gaps, and which questions they do and do not answer are described below.

### How these guidelines differ from others

Four methodological characteristics differentiate these guidelines from earlier versions. First, technical support was provided by a research librarian, Charlie Kishman, from the University of Cincinnati, who provided ongoing searches from eight distinct databases. Expertise in compiling data from a maximum of high-yield sources, and the thoroughness and methodology required to collate search terms and compile the results, impacts the quality guidelines or systematic reviews [2]. The convenience of having all relevant triaged articles land in an e-mail inbox (to be screened by team members from the pain and analgesia, agitation and sedation, delirium, and related clinical outcomes teams) cannot be overstated. The relevant articles were triaged in an online reference RefWorks site accessible to all members, for more manageable ongoing discussions and subsequent referencing. Second, all team members committed to focusing on published evidence in critically ill adults. No clinical experience-based opinion was incorporated in these guidelines, nor was any recommendation made where no evidence existed. Third, team member votes on the various recommendations were made public for readers to have access to the vote distribution. The intent, in addition to transparency and rigor, was to underscore that even in the context of rigorous evidence judgment plays a role in final attribution of scientific value and of importance. The GRADE method of assessment was chosen for ranking the evidence [3]. GRADE’s value as a barometer of evidence has not been demonstrated, nor has its reliability and agreement among experts [4]. Publishing the votes allows the guidelines reader to independently view the extent of concordance among voters grading the evidence contained within these guidelines. Fourth, the guidelines incorporate a variety of bedside behavioral assessment tools used to detect and evaluate pain, assess depth of sedation and degree of agitation, and detect delirium. No comparative assessments of the psychometric properties (i.e., scale reliability and validity) and feasibility of these tools for use in intensive care unit (ICU) patients are published. The assessment of the psychometric properties of applying pain, sedation and delirium scales to ICU patients [5] was undertaken, another first for this type of guideline.

Behavioral pain scales used in adult ICU patients were analyzed and compared adapting a previously published process [6]. Psychometric scoring systems were not available to evaluate and compare the psychometric properties of sedation and delirium scales, which have different validation strategies from those used for pain scales. The task force members undertook the development of similar psychometric scoring systems to assess and compare sedation and delirium scales, calling upon the expertise of psychometric experts and using accepted theoretical principles of health scale development and psychometric testing [5].

Conflict of interest declarations were left up to the individual authors; each author chose whether associations with different pharmaceutical industry partners constituted conflict. Experts with no conflicts are hard to find; some argued that being conflicted does not affect your judgment as to the quality of evidence [7]. Consensus among experts is subject to halo effects because of the group discussion process, where influence is challenging to differentiate from learning from each other and exchanging information. The strength of recommendations were ranked as strong (1) or weak (2), and either in favor of an intervention (+) or against (-) an intervention. For all strong recommendations, the phrase “*We recommend…*” was used. A weak recommendation indicated a less clear trade-off or weaker evidence; the phrase “*We suggest…*” was then used. In the absence of sufficient evidence, or when group consensus could not be achieved, no recommendation (0) was made.

### Pain assessment and analgesia

The current guidelines address several new dimensions of pain assessment and management described below. Several other areas remain unexplored, largely because of a dearth in publications; these also are summarized below.

### Pain in ICU patients

#### Incidence, consequences and outcomes

The current guidelines emphasize that pain is frequent in ICU patients [8], with an incidence of up to 50% in medical and surgical patients at rest [9], and increasing up to 80% during common care procedures. Since tracheal suctioning and drain removal as well as turning the patients for nursing-care procedures is reported as the most painful routine care procedures [10], routine preemptive analgesics in these circumstances are recommended. Pain in medical patients, which often is attributable to immobilization, can be addressed with early physiotherapy [11] and lighter sedation regimens as the current sedation and delirium portions of the guidelines suggest [1] (“*We recommend performing early mobilization of adult ICU patients whenever feasible to reduce the incidence and duration of delirium (+1B); and We recommend either daily sedation interruption or a light target level of sedation be routinely used in mechanically ventilated adult ICU patients (+1B)*”).

#### Long-term outcomes associated with pain or its management

The association between pain in ICU patients and the development of chronic pain syndrome in ICU survivors is not addressed [12] as studies published after December 2010 were not included in the evidence review and voting process. Posttraumatic stress disorder (PTSD) is attributed to pain memories in some studies [13-15]. Although one pediatric and one war-trauma study suggest more frequent use of morphine might reduce the risk of subsequent development of PTSD-related symptoms after injury [16,17], these findings have not been documented in an adult critical care population.

As these guidelines point out, improved pain management is clearly associated with better patient outcome in the ICU [8,18,19]. At least three studies performed in surgical, trauma, and medical ICUs report that a protocolized approach to assess and manage pain, agitation, and delirium [20] is associated with a reduced duration of mechanical ventilation, ICU acquired infections, length of stay and costs in ICU, and hospital as well as 30-day mortality [8,18-21]; accordingly, the guidelines recommend protocolized pain screening and assessing analgesic needs first to palliate the current under-recognition and treatment of pain [22].

#### Pain assessment

In patients able to communicate, self-report is without a doubt the most reliable method to assess pain [23]. The most commonly used are the Visual Analogue Scale (VAS), the Verbal Descriptor Scale (VDS) and the 0–10 Numeric Rating Scale (NRS). A prospective comparison of five popular self-report pain scales in terms of their feasibility, validity, and performance suggests that NRS-V is the most feasible and the pain scale preferred by patients [24]. Self-report pain tools were not rated by, but the NRS-V was considered preferable for pain detection in ICU patients. The thresholds for pain that usually trigger therapeutic interventions are defined by a V-NRS score greater than 3 points (scale range 0–10).

Since the review of pain measurement instruments available for ICU patients by Hamill-Ruth and Marohn [23] more than a decade ago highlighting the absence of validated instruments for critically ill patients, several new behavioral pain instruments have been described in the literature and were reviewed for guideline purposes [19,25-29]. Recent reviews [6,30] of these instruments concluded that only the Behavioral Pain Scale (BPS) [19] and the Critical Care Pain Observation Tool (CPOT) [27] provide acceptable levels of validity and reliability; these two behavioral scales now constitute the two recommended nonverbal pain screening techniques. The BPS was initially elaborated to assess pain in nonverbal, mechanically ventilated patients without severe head injury [19,31,32] with three behavioral domains: facial expression, upper limb movements, and compliance with ventilation. The CPOT has a fourth domain (muscle tension), which may be the most psychometrically valid of all in selected patients [27], such as the neurologically critically ill [33]. The BPS requires that ventilator asynchrony be observed while the patient undergoes a painful stimulus before attributing the asynchrony to pain, whereas the CPOT does not stipulate this requirement; this limitation is not addressed in the current guidelines. In addition, the CPOT has a vocalization domain to allow pain assessment in nonintubated patients, but this dimension’s psychometric properties have yet to be validated. More recently, a vocalization domain was added to the BPS, demonstrating good psychometric properties in ICU nonintubated patients [34]. Vital signs, such as heart rate and blood pressure, are unreliable as pain assessment surrogates in ICU patients compared with behavioral parameters [33-35]; the guidelines make this point and emphasize the need for systematic and rigorous pain assessment, particularly because ICU patients’ pain is consistently underrated by ICU caregivers [36-38]. Behavioral pain tools should not be used in communicative patients, because correlation coefficients between BPS and self-reported pain scales are low [24].

#### Treatment

No analgesic medication is associated with improved patient outcome. Opioids were used in up to 90% of mechanically ventilated patients in a multicenter patient-based study [39]. Accordingly, the guidelines “*…recommend that IV opioids should be considered as the first-line drug class of choice to treat nonneuropathic pain in critically ill patients (+1C)*.” How these opioids should be administered is not addressed. The continuous use of opioids may lead to drug and metabolite accumulation [40,41]. Intravenous perfusions are not mandatory; indeed, escalation of bolus opioids with or without continuous opiate infusion in ICU patients is feasible patients and associated with improved outcome [8,18,20].

A study performed in ICU patients (70% of whom were surgical) used a gradual escalation of analgesics from nonopioid drugs to incrementally powerful opioids [8]. In that study, tramadol use increased significantly, whereas incidence of pain and duration of mechanical ventilation decreased. Co-analgesia with nonopioids was introduced two decades ago [42] and is widely practiced for treating postoperative pain. In keeping with this rationale and despite the dearth of studies addressing co-analgesia effectiveness in critically ill patients, the guidelines suggest “*…that nonopioid analgesics be considered to decrease the quantity of opioids administered (or to eliminate the need for IV opioids altogether) and to decrease opioid-related side effects (+2C).*” In patients with end-stage liver disease, reduced dosing acetaminophen appears to be safe [43]. Nefopam, a centrally acting nonopioid agent proposed as an adjuvant to opioid analgesics, relieves moderate to severe pain in ICU patients without respiratory or neurological effects [44]. This description was published in 2011, after the final ratings and votes had been established for the publications being reviewed. Finally, gabapentin is effective for pain treatment opioid consumption reduction in ICU patients with Guillain-Barré syndrome [45]. This, and a similar study by the same group [46], led to the recommendation “…*that either enterally administered gabapentin or carbamazepine, in addition to intravenous IV opioids, should be considered for treatment of neuropathic pain (+1A)*.”

Regional analgesia (continuous epidural or peripheral nerve blocks) is known to improve the efficacy of traditional analgesic interventions and to decrease pulmonary complications in many postoperative patients [47]. Its integration into practice is variable over time and by European geographic region [48-50]. A multicenter study that included 1,416 continuous peripheral nerve blocks identified ICU stay as an independent factor associated with complications, such as hypoesthesia/paresthesia, local inflammation, and infection [50]. Use of regional analgesia in ICU patients deserves further evaluation in regards of its benefits, feasibility, side effects, and contraindications. Accordingly, the guidelines recommendations are cautious and limit their recommendation for regional anesthesia in the only population where it has been unequivocally demonstrated to be beneficial [51,52]. “*We recommend that thoracic epidural anesthesia/analgesia should be considered for postoperative analgesia in patients undergoing abdominal aortic surgery (+1B). We provide no recommendation for the use of a lumbar epidural over parenteral opioids for postoperative analgesia in patients undergoing abdominal aortic aneurysm surgery, due to a lack of benefit when these routes of administration are compared in this patient population (0,A). We provide no recommendation for the use of thoracic epidural analgesia in patients undergoing either intrathoracic or nonvascular abdominal surgical procedures, because of insufficient and conflicting evidence for this mode of analgesic delivery in these patients (0, B). We suggest that thoracic epidural analgesia may be considered for patients with traumatic rib fractures (+2B). We provide no recommendation for neuraxial/regional analgesia over systemic analgesia in medical ICU patients, due to lack of evidence in this patient population (0, No Evidence)*”.

### Critical care analgesia research perspectives: beyond the guidelines

The biology underpinning pain syndromes remains underexplored, particularly in medical ICU patients. Hyperalgesia associated with sepsis could contribute to a possible “diffuse ICU pain syndrome.” Indeed, myalgia and arthralgia are common clinical features associated with sepsis and fever [53], partly because of inflammation and muscle hypercatabolism induced by thermogenesis [54]. Inflammatory cytokines and sympathetic amines are associated with a nociceptive state associated with inflammation and sepsis [55,56].

Barriers associated with pain not being evaluated by health caregivers should be better elucidated [55,56]. In patients unable to communicate, electrophysiological measurements could allow for objective measurement of pain. The measurement of pupil size (pupillometry) appears to be more sensitive than behavioral parameters in a population of ICU patients undergoing nociceptive procedures [57]; whether pupillometry is useful compared with behavioral parameters during suctioning is less clear [58]. Which pain threshold should trigger analgesic administration is not well established. Randomized, controlled studies are still needed to demonstrate the association between pain assessment, analgesia, and short- and long-term outcomes (PTSD, chronic pain syndromes, and quality of life). Which pharmacologic interventions are useful, particularly with regards to co-analgesia, is uncertain. Surprisingly, there is a dearth of information on the use of acetaminophen/paracetamol or of anti-inflammatory agents in the ICU population [59,60].

Nonpharmacological adjuncts or substitutes to pharmacological intervention also are of interest. Music therapy and music are beneficial for critically ill patients. This unaddressed area in the literature is acknowledged in the current guidelines, which state: “*Complimentary, nonpharmacologic interventions for pain management, such as music therapy and relaxation techniques, can be considered as complementary therapies in pain management; they may be opioid-sparing and analgesia-enhancing choices, and they are low cost, easy to provide, and safe.*” Although a multimodal approach to pain management in ICU patients has been recommended, few studies have demonstrated the effectiveness of nonpharmacologic interventions [20,61,62].

### Sedation assessment and management

The current guidelines support the minimization of sedation so that patients are responsive and able to communicate. In clinical contexts where this is not possible or not desirable, daily interruption of sedation is encouraged. The recommendations take into account subsequently published data suggesting that interruption confers no additional advantage to sedation minimization [63]; although these publications were not available to the guidelines committee, the preliminary data were known and thought to be harmonious with the statements put forward earlier. These suggestions are novel compared with earlier guidelines, where the emphasis was on patient comfort but not necessarily of the harm inherent to sedatives. At the heart of earlier deliberations stood the conviction on the part of many caregivers that sedation mitigates how traumatic the patient perceives the ICU experience to be. This notion is slowly being contradicted by data from follow-up studies [64]. There is emerging understanding that excessive sedation, even when limited to 48 hours [65], is common and is associated with increased morbidity, mortality, and expenditure [65,66]. Daily interruption of sedative infusions, titration of sedative dose and opiates to symptoms [8,20,67], and minimization of drug administration is associated with patient benefit, reduced costs [21], and does not lead to accidental device removal or psychological stress [68]. The contrast to earlier guidelines is the explicit statement that harm is likely with iatrogenic coma; this point is made both in the sedation and delirium sections.

Despite review of the literature for the 10 years preceding the inception of the guidelines and during the 7 years spent in its creation, no clear recommendation could be put forward with regard to preferred sedation agent. Few topics generated as much controversy and discussion as the use or avoidance of benzodiazepines, leading to heated debates during panel discussions and to perspective-defining publications [69,70]. The answer to the question: *Should nonbenzodiazepine-based sedation, instead of sedation with benzodiazepines, be used in mechanically ventilated adult ICU patients?* (actionable) was reworded numerous times.

In parallel with the literature on continuously administered analgesic agents, few data report the pharmacokinetic properties of continuously administered benzodiazepines, propofol, and dexmedetomidine [71]. Any comparison of these agents should take into account the variability in half-life, terminal half-life, and changes associated with co-administration of competitive metabolic pathway agents, inflammatory status, and renal and hepatic dysfunction [70]. Dexmedetomidine was not labeled as preferable sedative agent except in delirious patients’ continuous sedation, where it is preferable to benzodiazepines. Cost also is a concern; benzodiazepines remain the least expensive molecule, albeit one subject to pharmacokinetic properties specific to the critically ill; the benefits of choosing any agent have to be considered in the light of the current context of health care cost containment policies in America and Europe [72,73].

### Delirium

Routine delirium assessment in all critically ill patients is recommended, in keeping with ICU guidelines published by others [74]. This shift in attributing importance to ICU delirium screening is integrated into regional or national accreditation requirements; for instance, ICU delirium screening is now mandated across Canada. Psychometric properties were assessed based on scale reliability and validity, and feasibility in critically ill adults. Scales were then rated on 1) item selection and content validation, 2) reliability, 3) validity, 4) feasibility, and 5) relevance or impact of implementation on patient outcomes, with regard to the scoring of delirium. The Intensive Care Delirium Screening Checklist (ICDSC) and the Confusion Assessment Method-ICU (CAM-ICU) were considered best. The presence of the original creators of these scales on the guidelines panel was not considered a conflict of interest; the psychometric validation was performed independently from their input. Two caveats should be considered: 1) the psychometric validation process, although performed by experts, was not peer-reviewed (by its publication or in any other form), in contrast to all other material considered for these guidelines; 2) despite application of rigorous psychometric validation principles, the fact that these two psychometrically valid scores detect ranges of delirium from 10% to >80% in similar populations was not accounted for.

The importance of delirium screening is emphasized to reassure patients and provide prognostic indicators. The recommendation that screening be performed with a tool rather than with clinical assessments by intensive care physicians was based on studies where delirium screening was introduced de novo [18] and by the strong opinions held by guidelines writers. A recent (unpublished until March 2013) study suggests, however, that clinical assessments by critical care physicians may identify delirium more rapidly and more accurately than screening tool assessments [75], a perhaps unsurprising finding. Delirium prevention is highlighted for the first time, with emphasis on early mobility as a safe and effective way of not only preventing delirium but providing patients with a more functional outcome at hospital discharge. Risk factors for ICU delirium are inconsistent across studies; those retained had to have been identified in at least two studies: these were preexisting dementia; history of hypertension; history of alcoholism; and admission a high severity of illness on admission. Some publications associate continuously administered benzodiazepine and delirium in critically ill patients [76,77].

Because continuously sedating patients with midazolam appears to be associated with a higher incidence of delirium than sedating patients with dexmedetomidine [78], and because this difference is not seen when morphine is compared to dexmedetomidine [79], midazolam has been presumed to be linked to delirium occurrence. A more recently published study suggests midazolam levels are in fact lower in patients who develop delirium [80]. The Confusion Assessment Method (CAM-ICU) screening tool was used to detect ICU delirium in studies that described less delirium with dexmedetomidine, a molecule that is associated with greater wakefulness than midazolam. Some authors have suggested that the CAM-ICU scoring may be affected by sedation [81]; the potential that the greater sedation seen and expected with midazolam was a confounder for delirium remains to be clarified before convincing conclusions can be drawn. With regard to more recently established risk factors for ICU delirium, the pre-deliric score [82] had not been published at the time of guideline writing; its risk stratification was not included in the guidelines document, and the risk prediction is currently being validated in an international multicenter study.

Delirium prophylaxis was addressed for the first time in these guidelines. To the question: “*Should a nonpharmacological delirium protocol in the ICU be used in the ICU to reduce the incidence or duration of delirium? (actionable)*,” the guidelines provide the following answer: “*We recommend that performing early mobilization of adult ICU patients be performed whenever feasible to reduce the incidence and duration of delirium (+1B)*.” This recommendation is based on the first multicenter, randomized, controlled trial of early mobility [11] and a subsequent implementation study, where investigators noted striking reductions in the incidence of delirium in mobilized patients. These studies also indicate that early and aggressive mobilization is unlikely to harm ICU patients, while reducing ICU and hospital LOS. Ongoing studies aiming to prove the benefit of multicomponent prevention [83] are currently under way.

No recommendations were made with regard to pharmacological delirium prevention. Six randomized studies evaluating a pharmacologic intervention for ICU patients had been published at the time of review. Although a reduction in delirium was observed in five of the studies, important methodological differences and limitations may have influenced some of the results [84,85], whereas other studies were limited to very narrow patient population [86,87]. Although use of dexmedetomidine as a sedative during elective cardiac surgery was associated with less delirium than propofol or midazolam [85], a subsequent study that was nearly four times larger found that use of dexmedetomidine resulted in a similar incidence of delirium compared with morphine-only sedation regimen [79]. In both of these sedation studies, delirium incidence was measured using the CAM–ICU; it remained unclear whether some of the patients deemed to have delirium actually had delirium or were simply sedated [88].

Given these data, no pharmacologic prophylaxis recommendation for delirium was made; a recent systematic review of delirium prophylaxis in the critically ill concurs with the lack of evidence to support this practice [89]. However, the reduction in subsyndromal delirium shown in one study was acknowledged as follows: “*One before/after study evaluated the impact of a multidisciplinary protocol for managing pain, agitation, and delirium in ICU patients*.” This study demonstrated a reduction in the incidence of *subsyndromal* delirium (but not delirium), with improved pain control, and without compromising sedation or anxiolysis, and a 15% reduction in their total ICU costs [20,21]. Subsyndromal delirium in ICU patients is defined as patients who have <4 points on the ICDSC; patients with subsyndromal delirium have worse clinical outcomes than those without delirium [90].

After the guidelines had been written, the largest ICU delirium prophylaxis study published to date described that a low-dose, 12-hour infusion of haloperidol reduces the incidence of delirium in surgical patients from 23% to 15% (*p* = 0.03) [91]. As the average APACHE scores were 9, the generalizability of these findings to the broad ICU population is not clear. However, encouraging preliminary single-center pre-post European pilot studies [92] have shown reductions in delirium incidence and duration in patients considered at high risk after prophylactic low doses of haloperidol, and support moving forward with multicenter prophylaxis trials in high-risk ICU populations. All successful prophylaxis studies to date have included surgical patients exclusively. In a well-conducted, but underpowered, multicenter, randomized, controlled pilot of delirium prophylaxis in medical ICU patients with antipsychotics (either haloperidol or ziprasidone vs. placebo) did not show any benefit with either haloperidol or ziprasidone compared with placebo [93].

The guidelines are clear as to the paucity of evidence supporting any pharmacologic treatment for delirium. With the possible exception of a benefit shown in a small (n = 36) study randomizing patients on as-needed haloperidol to quetiapine [94], where patients with quetiapine had shorter delirium duration, no drug has been shown to improve delirium outcome once it as occurred. Accordingly, the treatment section of the guidelines is clear: “Question: *Does treatment with haloperidol reduce the duration of delirium in adult ICU patients? (descriptive)* Answer: *There is no published evidence that treatment with haloperidol reduces the duration of delirium in adult ICU patients (No Evidence),* and Question: *Does treatment with atypical antipsychotics reduce the duration of delirium in adult ICU patients? (descriptive)* Answer: *Atypical antipsychotics may reduce the duration of delirium in adult ICU patients (C)*.” A recent review of delirium treatment in ICU patients [95] concedes that there is limited evidence on the safety and effectiveness of antipsychotics in ICU delirium.

Rivastigmine use in the critically ill is specifically discouraged in the guidelines: “*We do not recommend administering rivastigmine to reduce the duration of delirium in ICU patients (-1B)*.” Although tested in only one study, when rivastigmine was compared with placebo in critically ill patients the investigation was halted because of perceived futility and potential harm by the DSMB (Data Safety Monitoring Board) [96]; rivastigmine-treated patients were found to have more severe and longer delirium, with a trend toward a higher mortality rate. Delirium in association to alcohol withdrawal was not covered in these guidelines because of space considerations; this topic is reviewed in a separate publication [95].

## Conclusions

The current guidelines update the critical care clinician on the importance of pain and delirium assessments and the need to minimize or interrupt sedation. The methodological rigor with which the evidence was triaged improves the clarity and scientific basis of the recommendations; the guidelines also better identify gaps in current evidence. We hope that the current update on guideline content and process will incite critical care investigators to build on established work to address pain, sedation, and delirium issues in order to improve care and outcomes for the critically ill and to reduce the significant burden of critical illness on patients, their families, and society.

## Competing interests

The authors declare that they have no competing interests.

## Authors’ contributions

YS was part of the Society of Critical Care Medicine’s guidelines committee. Both authors wrote sections of the manuscript, and extensively reviewed its entire content. Both authors read and approved the final manuscript.
